# Vascular and Microvascular Dysfunction Induced by Microgravity and Its Analogs in Humans: Mechanisms and Countermeasures

**DOI:** 10.3389/fphys.2020.00952

**Published:** 2020-08-20

**Authors:** Nastassia Navasiolava, Ming Yuan, Ronan Murphy, Adrien Robin, Mickael Coupé, Linjie Wang, Asmaa Alameddine, Guillemette Gauquelin-Koch, Claude Gharib, Yinghui Li, Marc-Antoine Custaud

**Affiliations:** ^1^Clinical Research Center, CHU d’Angers, Angers, France; ^2^State Key Laboratory of Space Medicine Fundamentals and Application, China Astronaut Research and Training Center (ACC), Beijing, China; ^3^School of Health and Human Performance, Faculty of Science & Health, Dublin City University, Dublin, Ireland; ^4^Mitovasc, UMR INSERM 1083-CNRS 6015, Université d’Angers, Angers, France; ^5^Centre National d’Études Spatiales (CNES), Paris, France; ^6^Institut NeuroMyoGène, Faculté de Médecine Lyon-Est, Université de Lyon, Lyon, France

**Keywords:** vascular deconditioning, endothelium, vascular remodeling, vascular risk, prevention, shear stress

## Abstract

Weightlessness and physical inactivity have deleterious cardiovascular effects. The space environment and its ground-based models offer conditions to study the cardiovascular effects of physical inactivity in the absence of other vascular risk factors, particularly at the macro- and microcirculatory levels. However, the mechanisms involved in vascular dysfunction and remodeling are not sufficiently studied in the context of weightlessness and its analogs including models of physical inactivity. Here, we summarize vascular and microvascular changes induced by space flight and observed in models of microgravity and physical inactivity and review the effects of prophylactic strategies (i.e., countermeasures) on vascular and microvascular function. We discuss physical (e.g., exercise, vibration, lower body negative pressure, and artificial gravity) and nutritional/pharmacological (e.g., caloric restriction, resveratrol, and other vegetal extracts) countermeasures. Currently, exercise countermeasure appears to be the most effective to protect vascular function. Although pharmacological countermeasures are not currently considered to fight vascular changes due to microgravity, nutritional countermeasures are very promising. Dietary supplements/natural health products, especially plant extracts, should be extensively studied. The best prophylactic strategy is likely a combination of countermeasures that are effective not only at the cardiovascular level but also for the organism as a whole, but this strategy remains to be determined.

## Introduction

A host of physiological alterations occur in actual or simulated microgravity, including fluid changes, hormonal changes, muscle atrophy and force reduction, bone loss, autonomic dysregulation, cardiac atrophy, vascular impairment, and microcirculatory dysfunction (Coupé et al., 2009; Evans et al., 2018; Hughson et al., 2018). Surprisingly, the cardiovascular system of astronauts adapts well to microgravity. However, the price of this adaptation is rapid cardiovascular deconditioning – a syndrome combining orthostatic intolerance, increased heart rate, and decreased exercise capacity, accompanied by vascular disorders. Similar cardiovascular deconditioning is also observed on Earth and was first described in bedridden patients in 1945 (Keys, 1945). Physical inactivity is one of the key factors contributing to this cardiovascular deconditioning.

Sedentariness is among the most important behavioral risk factors for cardiovascular diseases (CVD). In terms of the cardiovascular system, the risk of being unfit exceeds the risks associated with smoking, elevated blood pressure, hypercholesterolemia, or obesity, whereas regular exercise is associated with a reduction in vascular events (Thijssen et al., 2010). Studying physical inactivity in healthy individuals in the absence of other vascular risk factors provides valuable information concerning vascular diseases. Because fighting gravity requires daily physical exercise, exposure to microgravity is associated with enhanced inactivity (Hughson, 2009; Hughson et al., 2018). Microgravity and its analogs – bed rest, head-down bed rest (HDBR) (Fortney et al., 1996; Pavy-Le Traon et al., 2007), and dry immersion (Navasiolava et al., 2011; Watenpaugh, 2016; Tomilovskaya et al., 2019) – offer unique models for studying the effects of global physical inactivity in healthy individuals (Widlansky, 2010). However, other models of pure physical inactivity are also valuable, such as remaining in a sitting position for a few hours (Restaino et al., 2015; Thosar et al., 2015; Morishima et al., 2016) or reducing walking to below 5,000 steps/day, which is easy to implement and is shown to impair vascular and metabolic functions (Boyle et al., 2013; Teixeira et al., 2017). Segmental inactivity models are also used, with unilateral lower limb suspension (Bleeker et al., 2005a, c) and limb casting (Green et al., 1997; Sugawara et al., 2004) being less extreme models. Although confinement has also been considered a model of spaceflight (Arbeille et al., 2014; Yuan et al., 2019), its associated inactivity is difficult to attest.

Several countermeasures to prevent microgravity- and inactivity-induced cardiovascular changes, including exercise, whole body vibration (WBV), lower body negative pressure (LBNP), centrifugation, caloric restriction, nutritional supplements, natural health products, and medications, have been evaluated. The protection of astronaut health is a critical issue in space medicine. However, knowledge acquired in this area could be extended to the general sedentary population.

Here, we will summarize the effects of microgravity and its analogs on macro- and microcirculation in humans. We will also review the effects of different countermeasures proposed in the context of microgravity and its analogs on vascular and microvascular functions.

## Vascular and Microvascular Changes Induced by Physical Inactivity and Microgravity

### Conduit Artery Changes

Data on lower limb, brachial, and carotid arterial changes in modeled and actual microgravity are summarized in Tables 1–4.

**TABLE 1:** Lower limb conduit arteries: effects of experimental models and countermeasures.

Variable	Inactivity model	Duration	Subjects Ctrl/CM	Vascular level	Estimated inactivity effect (without CM)	CM tested	Estimated CM effects	References
Diameter	Sitting	3 h	7M + 4F	Popliteal	No effect	Unilateral fidgeting bouts	No effect	Morishima et al. (2016)
	< 5,000 steps/d	5 d	11M	Popliteal	No effect	_	_	Boyle et al. (2013)
	< 5,000 steps/d	5 d	13M	Popliteal	↓2% (NS)	Unilateral foot heating bouts	Partly prevented (↓1% (NS))	Teixeira et al. (2017)
	Unilateral leg cast	7 d	8M	Femoral	↓5%	Contralateral leg	Completely prevented	Sugawara et al. (2004)
	Unilateral leg suspension	28 d	3M + 4F	Femoral	↓12%	Contralateral leg	Completely prevented	Bleeker et al. (2005a)
	Horizontal BR	25 d	8M/8M	Femoral	↓13%	RVE	Partially prevented (↓5%)	Bleeker et al. (2005b)
	HDBR	35 d	10M	Femoral	↓10%	_	_	Palombo et al. (2015)
	Horizontal BR	52 d	8M/8M	Femoral	↓17%	RVE	Partially prevented (↓6%)	Bleeker et al. (2005b)
	HDBR	56 d	7F/8F	Femoral	No effect	LBNP + aerobic + RE	No effect	Zuj et al. (2012b)
	HDBR	60 d	7M/7M	Femoral	↓33%	CHM	No effect	Yuan et al. (2015)
	HDBR	60 d	9M/9M	Femoral	↓24%	RE	No effect	van Duijnhoven et al. (2010a)
	HDBR	60 d	9M/9M	Femoral	↓24%	RVE	Partially prevented	van Duijnhoven et al. (2010a)
IMT	Unilateral leg cast	7 d	8M	Femoral	No effect	Contralateral leg	No effect	Sugawara et al. (2004)
	HDBR	35 d	10M	Femoral	No effect	_	_	Palombo et al. (2015)
	HDBR	60 d	9M/9M	Femoral	↑12%	RE	Prevented	van Duijnhoven et al. (2010a)
	HDBR	60 d	9M/9M	femoral	↑12%	RVE	Prevented	van Duijnhoven et al. (2010a)
Stiffness	Unilateral leg cast	7 d	8M	Femoral	↓ (NS)	Contralateral leg	Completely prevented	Sugawara et al. (2004)
	HDBR	35 d	10M	Femoral	no effect	_	_	Palombo et al. (2015)
	HDBR	60 d	7M/7M	Femoral	↑	CHM	No effect	Yuan et al. (2015)
Flow-mediated dilation	Sitting	3 h	12M crossover	Femoral	↓2-3%	Three bouts of 5-min walking	Completely prevented	Thosar et al. (2015)
	Sitting	3 h	7M+4F	popliteal	↓3%	Unilateral fidgeting bouts	↑3%	Morishima et al. (2016)
	Sitting	6 h	11M	Popliteal	↓4%	10-min walk post-sitting	Restored	Restaino et al. (2015)
	<5,000 steps/d	5 d	11M	Popliteal	↓3%	_	_	Boyle et al. (2013)
	< 5,000 steps/d	5 d	13M	Popliteal	↓4%	Unilateral foot heating bouts	Completely prevented	Teixeira et al. (2017)
	Unilateral leg suspension	28 d	3M + 4F	Femoral	↑3–4%	_	_	Bleeker et al. (2005a)
	Horizontal BR	25 d	8M/8M	Femoral	↑4%	RVE	Completely prevented	Bleeker et al. (2005b)
	HDBR	49 d	8M+5F	Tibial	↑5%	_	_	Platts et al. (2009)
	Horizontal BR	52 d	8M/8M	Femoral	↑4%	RVE	No effect	Bleeker et al. (2005b)
	HDBR	60 d	9M/9M	Femoral	↑7%	RE	Partially prevented (NS)	van Duijnhoven et al. (2010b)
	HDBR	60 d	9M/9M	Femoral	↑7%	RVE	Completely prevented	van Duijnhoven et al. (2010b)
Nitroglycerin-mediated dilation	Unilateral leg suspension	28 d	3M+4F	Femoral	↑4–5%	_	_	Bleeker et al. (2005a)
	Horizontal BR	25 d	8M/8M	Femoral	No effect	RVE	No effect	Bleeker et al. (2005b)
	HDBR	49 d	8M + 5F	Tibial	↑6–7% (NS)	_	_	Platts et al. (2009)
	Horizontal BR	52 d	8M/8M	Femoral	↑4%	RVE	No effect	Bleeker et al. (2005b)
	HDBR	56 d	7F/8F	Femoral	No effect	LBNP + aerobic + RE	No effect	Zuj et al. (2012b)

*BR, bed rest; CHM, Chinese herbal medicine; CM, countermeasure; RE, resistive exercise; RVE, resistive vibration exercise; F, female; M, male; NS, not significant.*

**TABLE 2:** Common carotid artery: effects of experimental models and countermeasures.

Variable	Inactivity model	Duration	Subjects Ctrl/CM	Estimated inactivity effect (without CM)	CM tested	Estimated CM effects	References
Diameter	HDBR	7 d	8M crossover	↓5% (NS)	Thigh cuffs	No effect	Arbeille et al. (1999)
	HDBR	35 d	10M	No effect	_	_	Palombo et al. (2015)
	Horizontal BR	52 d	8M/8M	No effect	RVE	No effect	Bleeker et al. (2005b)
	HDBR	60 d	7M/7M	No effect	CHM	No effect	Yuan et al. (2015)
	HDBR	60 d	9M/9M	No effect	RE	No effect	van Duijnhoven et al.(2010a,b)
	HDBR	60 d	9M/9M	No effect	RVE	No effect	van Duijnhoven et al.(2010a,b)
IMT	Confinement	180 d	3M+1F	↑10–15%	_	_	Yuan et al. (2019)
	Confinement	520 d	6M	↑14–28%	_	_	Arbeille et al. (2014)
	HDBR	35 d	10M	No effect	_	_	Palombo et al. (2015)
	HDBR	60 d	7M/7M	No effect	CHM	No effect	Yuan et al. (2015)
	HDBR	60 d	9M/9M	↑17%	RE	Completely prevented	van Duijnhoven et al. (2010a)
	HDBR	60 d	9M/9M	↑17%	RVE	Completely prevented	van Duijnhoven et al. (2010a)
Stiffness	Confinement	180 d	3M+1F	No effect	_	_	Yuan et al. (2019)
	HDBR	35 d	10M	No effect	_	_	Palombo et al. (2015)
	HDBR	60 d	7M/7M	No effect	CHM	No effect	Yuan et al. (2015)

*BR, bed rest; CHM, Chinese herbal medicine; CM, countermeasure; RE, resistive exercise; RVE, resistive vibration exercise; F, female; M, male; NS, not significant.*

**TABLE 3:** Brachial artery: effects of experimental models and countermeasures.

Variable	Inactivity model	Duration	Subjects Ctrl/CM	Estimated inactivity effect (without CM)	CM tested	Estimated CM effects	References
Diameter	<5,000 steps/d	5 d	11M	↓5%	_	_	Boyle et al. (2013)
	Horizontal BR	5 d	14M+6F	↓2–3%	_	_	Hamburg et al. (2007)
	HDBR	7 d	8M/8M	No effect	Usual daily activity	No effect	Bonnin et al. (2001)
	Horizontal BR	52 d	8M/8M	↓6%	RVE	No effect	Bleeker et al. (2005b)
	HDBR	56 d	16F/8F	No effect	LBNP + aerobic + RE	No effect	Hughson et al. (2007)
IMT	HDBR	49 d	8M+5F	No effect	_	_	Platts et al. (2009)
Flow-mediated dilation	Sitting	6 h	11M	No effect	10-min walk post-sitting	No effect	Restaino et al. (2015)
	<5,000 steps/d	5 d	11M	no effect	_	_	Boyle et al. (2013)
	Horizontal BR	5 d	14M + 6F	No effect	_	_	Hamburg et al. (2007)
	HDBR	7 d	8M/8M	↑5%	Usual daily activity	Completely prevented	Bonnin et al. (2001)
Nitroglycerin-mediated dilation	Horizontal BR	5 d	14M+6F	No effect	_	_	Hamburg et al. (2007)
	HDBR	7 d	8M/8M	No effect	Usual daily activity	No effect	Bonnin et al. (2001)
	HDBR	49 d	8M+5F	No effect	_	_	Platts et al. (2009)

*BR, bed rest; CM, countermeasure; RE, resistive exercise; RVE, resistive vibration exercise; F, female; M, male.*

**TABLE 4:** Vascular changes induced by spaceflight.

Vascular level	Variable	Measurement timepoints	*n*	Flight effect	References
Central and peripheral vessels	Heart-finger pulse transit time	6 mo	8	↓5–6%	Hughson et al. (2016)
	Heart-ankle pulse transit time	6 mo	8	↓2–3%	Hughson et al. (2016)
Carotid	IMT	d15	10	↑10%	Arbeille et al. (2016)
	IMT	d115–165	10	↑12%	Arbeille et al. (2016)
	IMT	d15, d60, and d160	13	No effect	Lee et al. (2020)
	IMT	m1, m2, m6, m8, and m10	1	↑∼20%	Garrett-Bakelman et al. (2019)
	Diameter	d15 and d115–165	10	No effect	Arbeille et al. (2016)
	Diameter	m1, m2, m6, m8, and m10	1	↑∼7%	Garrett-Bakelman et al. (2019)
	Diameter	d15, d60, and d160	13	↑∼5%	Lee et al. (2020)
	Stiffness	pre/post 6-mo flight	8	↑17–30%	Hughson et al. (2016)
	Stiffness	Inflight 6-mo flight	10		Arbeille et al. (2017)
	Stiffness	d15, d60, and d160	13	No effect	Lee et al. (2020)
Upper limb	Brachial diameter	d15, d60, and d160	13	No effect	Lee et al. (2020)
	Brachial FMD	d15, d60, and d160	13	No effect	Lee et al. (2020)
	Brachial nitroglycerin-mediated dilation	d15, d60, and d160	13	No effect	Lee et al. (2020)
	Pulse blood filling	6 mo	11	↓12%	Turchaninova et al. (2001)
	Conduit arteries vascular tone (rheography)	6 mo	11	↑23%	Turchaninova et al. (2001)
	Pre-capillary vascular tone (rheography)	6 mo	11	↓60%	Turchaninova et al. (2001)
Lower limb	Femoral IMT	d15	10	↑10%	Arbeille et al. (2016)
	Femoral IMT	d115–165	10	↑15%	Arbeille et al. (2016)
	Femoral diameter	d15, d115–165	10	No effect	Arbeille et al. (2016)
	Femoral stiffness	d15, d115–165	10	↑20–30% (NS)	Arbeille et al. (2017)
	Resting vasular resistance (ultrasound)	1–6 mo	7	↓10%	Arbeille et al. (2001, 1995)
	Vasular resistance response to LBNP (ultrasound)	6 mo	7	↓(+40% pre vs +15% inflight)	Herault et al. (2000)
	Resting blood flow (ultrasound)	1–6mo	7	No effect	Arbeille et al. (2001)
	Resting blood flow (plethysmography)	d4–12	7	↓41%	Watenpaugh et al. (2001)
	Vascular resistance (plethysmography)	d4–12	7	↑93%	Watenpaugh et al. (2001)
	Pulse blood filling	6 mo	11	↓19%	Turchaninova et al. (2001)
	Conduit arteries vascular tone (rheography)	6 mo	11	No effect	Turchaninova et al. (2001)
	Pre-capillary vascular tone (rheography)	6 mo	11	No effect	Turchaninova et al. (2001)

#### Physical Inactivity Induces Structural Changes in Conduit Arteries

Physical inactivity has a marked effect on the structure of conduit arteries. For lower limb arteries, particularly unloaded in our models, physical inactivity is associated with inward remodeling and decreased lumen diameter to differing degrees (Sugawara et al., 2004; Bleeker et al., 2005a, b; de Groot et al., 2006; van Duijnhoven et al., 2010a; Palombo et al., 2015; Yuan et al., 2015) depending on intensity and duration of inactivity (Thijssen et al., 2010). Intima media thickness (IMT) at the femoral artery level remains unchanged after short-term inactivity, such as 7-day leg casting (Sugawara et al., 2004) and after 35-day HDBR (Palombo et al., 2015), but increases with longer inactivity, such as 60-day HDBR (van Duijnhoven et al., 2010a). At the carotid artery level, diameter remains unchanged (Arbeille et al., 1999; Bleeker et al., 2005b; van Duijnhoven et al., 2010a, b; Palombo et al., 2015; Yuan et al., 2015). For brachial artery, which is generally not unloaded in our models, diameter remains stable (Bonnin et al., 2001; de Groot et al., 2004; Hughson et al., 2007) or is very slightly decreased (Boyle et al., 2013; Bleeker et al., 2005b; Hamburg et al., 2007). IMT at the carotid level is unmodified (Palombo et al., 2015; Yuan et al., 2015) or increased (van Duijnhoven et al., 2010a) after long-term HDBR. Long-term confinement also increases carotid IMT as evidenced from Mars-520d (Arbeille et al., 2014) and CELSS-180d (Yuan et al., 2019) experiments. Concerning macrovascular elasticity at the femoral level, 35-day HDBR does not affect compliance (Palombo et al., 2015), whereas 7-day leg casting tends to decrease (Sugawara et al., 2004) and 60-day HDBR decreases compliance (Yuan et al., 2015). Carotid compliance is not modified by HDBR (Palombo et al., 2015; Yuan et al., 2015) or confinement (Yuan et al., 2019).

Regarding flight findings, a 6-month mission did not modify femoral or carotid diameter but rapidly and steadily increased carotid IMT up to 10–12% (Arbeille et al., 2016). Similarly, in the National Aeronautics and Space Administration twin study with one astronaut and one ground control, a 1-year mission increased carotid IMT by ∼20% (Garrett-Bakelman et al., 2019). However, Lee et al. (2020) did not observe changes in carotid IMT and stiffness at d15, d60, and d160 of long-term flight, while carotid diameter was increased (+5% during diastole). Lee et al. consider possibility that increase in IMT might be hidden by carotid distention. Findings from several inflight studies suggest that most astronauts represent increase in IMT. Presently no environmental (physical activity, nutrition, stress…) or genetic factor has been identified as a major factor related to this increase. Femoral IMT increases up to 10–15% (Arbeille et al., 2016; Hughson et al., 2018). Carotid (Hughson et al., 2016; Arbeille et al., 2017; Hughson et al., 2018) and femoral (Arbeille et al., 2017; Hughson et al., 2018) stiffness are also increased. Moreover, a 6-month flight reduced pulse transit time, suggesting stiffer central and peripheral arteries (Hughson et al., 2018). Limb conduit artery tone, as estimated by rheography, increased in a 6-month flight at the forearm but not at the calf level (Turchaninova et al., 2001).

#### Physical Inactivity and the Reactivity of Conduit Arteries

At the leg level, brief inactivity, such as that induced by a few hours of sitting, decreases endothelium-dependent vasodilation capacity as estimated by flow mediated dilation (FMD) (Restaino et al., 2015; Thosar et al., 2015; Morishima et al., 2016). Similarly, reducing daily physical activity by taking <5,000 steps/day decreases FMD response at the popliteal level, whereas basal popliteal diameter is unchanged (Boyle et al., 2013; Teixeira et al., 2017). In both cases, intermittent application of maneuvers increasing blood flow, such as fidgeting (Morishima et al., 2016) or foot heating to 42°C (Teixeira et al., 2017), preserves popliteal FMD. However, prolonged advanced inactivity does not change (de Groot et al., 2004) or even increases (Bleeker et al., 2005a, b; de Groot et al., 2006; Platts et al., 2009; van Duijnhoven et al., 2010b) FMD of leg arteries, which may be explained by inward remodeling of arterial vessels at the lower limb level. Smooth muscle vasodilator functions of large arteries are not impaired and may be enhanced after physical inactivity. At the brachial level, no significant changes in sensitivity to nitric oxide (NO) are observed after 5, 7, 49, or 56 days of bed rest (Bonnin et al., 2001; Hamburg et al., 2007; Platts et al., 2009). Changes at the lower limb level are less homogenous. Vasodilation in response to nitroglycerin is unmodified after 25 days of horizontal bed rest (Bleeker et al., 2005b) or 56 days of HDBR (Zuj et al., 2012b), tends to increase after 49 days of HDBR (Platts et al., 2009), and increases after 28 days of unilateral lower limb suspension (Bleeker et al., 2005a) or 52 days of horizontal bed rest (Bleeker et al., 2005b).

As for inflight reactivity of large arteries, endothelium-dependent and –independent vasodilation at brachial artery level is not substantially modified with long-term flight, while brachial diameter is unchanged (*n* = 13, measurements at d15, d60, d160; Lee et al., 2020).

### Resistance Vessels and Microcirculation

#### Systemic Vascular Resistance

Total peripheral resistance characterizes in a integrative way the global resistance of all the systemic vasculature. In most HDBR and dry immersion studies, systemic vascular resistance is increased (Pavy-Le Traon et al., 2007; Navasiolava et al., 2011). By contrast, in inflight studies, systemic vascular resistance seems to be decreased as indirectly deduced from a measured decrease in blood pressure and increase in cardiac output, despite preserved or even increased sympathetic nervous activity (Norsk, 2019). Mechanisms for this unexpected systemic vasodilation remain to be identified. Proposed contributors are a headward fluid shift-induced decrease in lower body vessel stretch (with reduced myogenic tone), cardiac distention (with release of cardiac vasodilatory natriuretic peptides), and increased core body temperature (Norsk, 2019).

#### Regional and Organ Circulations

Data on changes at arteriolar and microcirculatory level in actual and modeled microgravity are summarized in Tables 4, 5.

**TABLE 5:** Regional limb circulation: effects of experimental models and countermeasures.

Vascular level	Variable	Inactivity model	Duration	Subjects Ctrl/CM	Estimated inactivity effect (without CM)	CM tested	Estimated CM effects	References
Lower limb	Resting blood flow (plethysmography)	Unilateral leg suspension	28 d	3M + 4F	↓24%	Contralateral leg	Completely prevented	Bleeker et al. (2005a)
	Resting blood flow (plethysmography)	HDBR	41 d	7M	↓49%	_	_	Louisy et al. (1997)
	Resting blood flow (ultrasound)	Horizontal BR	52 d	8M	No effect	RVE	no effect	Bleeker et al. (2005b)
	Resting blood flow (ultrasound)	HDBR	4, 7, and 42 d	8M	No effect	_	_	Arbeille et al. (2001)
	Resting blood flow (plethysmography)	HDBR	118 d	6M	↓43%	_	_	Christ et al. (2001)
	Vascular resistance (plethysmography)	HDBR	14 d	20M	↑50%	_	_	Kamiya et al. (2000)
	Vascular resistance (plethysmography)	HDBR	18 d	11M + 1F	↑35%	_	_	Pawelczyk et al. (2001)
	Vascular resistance (ultrasound)	HDBR	7 and 42 d	8M	↓10–20%	_	_	Arbeille et al. (2001)
	Vascular resistance (plethysmography)	Horizontal BR	20 d	6M+3F	No effect	_	_	Bonde-Petersen et al. (1994)
	Constriction to incremental L-NMMA (NO synthase inhibitor) (plethysmography)	Unilateral leg suspension	28 d	3M+4F	No effect	_	_	Bleeker et al. (2005c)
	Dilation post-occlusion (ultrasound)	Sitting	6 h	11M	↓43%	10-min walk post-sitting	restored	Restaino et al. (2015)
	Dilation post-occlusion (plethysmography)	Horizontal BR	5 d	10/9	↓22%	usual activity	completely prevented	Hamburg et al. (2007)
	Dilation to incremental SNP (plethysmography)	Unilateral leg suspension	28 d	3M + 4F	No effect	_	_	Bleeker et al. (2005c)
	Vasoconstriction to LBNP (ultrasound)	HDBR	28 d	6M/6M	Impaired vasoconstrictive response	Exercise + LBNP	Completely prevented	Arbeille et al. (1995)
	Vasoconstriction to LBNP (ultrasound)	HDBR	55 d	8F/8F	Impaired vasoconstrictive response	LBNP + aerobic + RE	Completely prevented	Arbeille et al. (2008)
Calf skin	Resting blood flow (laser Doppler)	Dry immersion	7 d	8M	↓30–40%	_	_	Navasiolava et al. (2010)
	Resting blood flow (laser Doppler)	HDBR	56 d	8F/8F	↓20% (NS)	LBNP + aerobic + RE	Completely prevented	Demiot et al. (2007)
	Resting blood flow (laser Doppler)	HDBR	60 d	7M/7M	↓30% (NS)	CHM	No effect	Yuan et al. (2015)
	Max dilation to heating (laser Doppler)	Dry immersion	7 d	8M	No effect	_	_	Navasiolava et al. (2010)
	Max dilation to heating (laser Doppler)	HDBR	56 d	8F/8F	No effect	LBNP + aerobic + RE	No effect	Demiot et al. (2007)
	Max dilation to heating (laser Doppler)	HDBR	60 d	7M/7M	No effect	CHM	No effect	Yuan et al. (2015)
	Dilation to ACh (laser Doppler)	Dry immersion	7 d	8M	↓17%	_	_	Navasiolava et al. (2010)
	Dilation to ACh (laser Doppler)	HDBR	56 d	8F/8F	↓11%	LBNP + aerobic + RE	Completely prevented	Demiot et al. (2007)
	Dilation to ACh (laser Doppler)	HDBR	60 d	7M/7M	↓15%	CHM	Completely prevented	Yuan et al. (2015)
	Dilation to SNP (laser Doppler)	Dry immersion	7 d	8M	No effect	_	_	Navasiolava et al. (2010)
	Dilation to SNP (laser Doppler)	HDBR	56 d	8F/8F	No effect	LBNP + aerobic + RE	No effect	Demiot et al. (2007)
	Dilation to SNP (laser Doppler)	HDBR	60 d	7M/7M	No effect	CHM	No effect	Yuan et al. (2015)
Upper limb	Resting blood flow (plethysmography)	HDBR	13 d	10M	↓19% (NS)	Low fat hypoenergetic diet (crossover)	No effect	Hesse et al. (2005)
	Resting blood flow (plethysmography)	HDBR	14 d	8/12	↓15%	Cycle ergometry	No effect	Crandall et al. (2003)
	Resting blood flow (plethysmography)	Forearm cast	42 d	6M/6M	No effect	Non-casted controls	No effect	Green et al. (1997)
	Resting blood flow (ultrasound)	Horizontal BR	52 d	8M/8M	No effect	RVE	No effect	Bleeker et al. (2005b)
	Resting resistance (plethysmography)	HDBR	18 d	11M + 1F	No effect	_	_	Pawelczyk et al. (2001)
	Resting resistance (plethysmography)	Horizontal BR	20 d	6M + 3F	No effect	_	_	Bonde-Petersen et al. (1994)
	Dilation to incremental ACh (plethysmography)	HDBR	13 d	10M	↓	Low fat hypoenergetic diet (crossover)	Completely prevented	Hesse et al. (2005)
	Constriction to L-NMMA (NO synthase inhibitor) (plethysmography)	Forearm cast	42 d	6M/6M	no effect	Non-casted controls	No effect	Green et al. (1997)
	Dilation post-occlusion (ultrasound)	Sitting	6 h	11M	↓31%	10-min walk post-sitting	No effect	Restaino et al. (2015)
	Dilation post-occlusion (ultrasound)	Horizontal BR	5 d	14M+6F/9	↓16%	Usual activity		Hamburg et al. (2007)
	Dilation post-occlusion (plethysmography)	HDBR	14 d	20M	↓30%	_	_	Shoemaker et al. (1998)
	Dilation to incremental SNP (plethysmography)	HDBR	13 d	10M	No effect	Low fat hypoenergetic diet (crossover)	No effect	Hesse et al. (2005)
	Dilation to heating (plethysmography)	HDBR	14 d	8/12	↓13%	Cycle ergometry	Completely prevented	Crandall et al. (2003)
Forearm skin	Dilation to ACh (laser Doppler)	Confinement	180 d	3M+1F	↓17%	_	_	Yuan et al. (2019)

*BR, bed rest; CHM, Chinese herbal medicine; CM, countermeasure; RE, resistive exercise; RVE, resistive vibration exercise; F, female; M, male; NS, not significant.*

##### Lower- and upper limb vascular resistance

Limb vascular resistance can be measured directly at rest by Doppler ultrasound and estimated indirectly during inflation-deflation dynamic test by plethysmography. The assessment method for each result is indicated in Tables 4, 5. At the lower limb level, most plethysmographic studies demonstrate a decrease in resting blood flow (Louisy et al., 1997; Christ et al., 2001; Bleeker et al., 2005a; Navasiolava et al., 2010) and increase in vascular resistance after inactivity (Kamiya et al., 2000; Pawelczyk et al., 2001), although some do not detect changes (Bonde-Petersen et al., 1994; Bleeker et al., 2005b). At the upper limb level, resting blood flow and resistance remain mostly unmodified by inactivity (Bonde-Petersen et al., 1994; Green et al., 1997; Pawelczyk et al., 2001; Bleeker et al., 2005b), although some studies report a decrease in resting flow (Crandall et al., 2003; Hesse et al., 2005). Furthermore, a lack of increase in lower limb vascular resistance (insufficient vasoconstriction) in response to LBNP was observed following 60-day HDBR in women, together with unmodified total sympathetic nerve activity as assessed by microneurography, suggesting that deconditioning concerned rather the distal vascular tree targets (vasomotor response) than autonomic regulation (Arbeille et al., 2008).

Regarding flight findings, calf resting flow decreased and vascular resistance increased early inflight (days 4–12), as estimated by plethysmography (Watenpaugh et al., 2001). On the other hand, direct measurement from femoral arterial flow revealed significant decrease of lower limb vascular resistance in 14-day and 6-month flight (Arbeille et al., 1995, 2001). A lack of increase in lower limb vascular resistance (vasoconstriction) in response to LBNP at 1st and 5th month of flight was documented, as measured by Doppler ultrasound (Herault et al., 2000), similar to what observed in HDBR.

Rheography in a 6-month mission revealed pulse blood filling decrease at the forearm (−12%) and shin (−19%) levels. Also, pre-capillary vascular tone decreased at the forearm but not at the calf level (Turchaninova et al., 2001).

##### Cerebral circulation and question of spaceflight associated neuro-ocular syndrome (SANS)

Data concerning microgravity effect on cerebral autoregulation are controversial. Indeed, bed rest has been reported to impair (Zhang et al., 1997; Sun et al., 2005), or to maintain (Pavy-Le Traon et al., 2002), or to improve (Jeong et al., 2014; Kermorgant et al., 2019) cerebral autoregulation. Similarly, in astronauts cerebral autoregulation might be impaired (Zuj et al., 2012a), preserved (Herault et al., 2000; Arbeille et al., 2001; Iwasaki et al., 2007), or improved (Iwasaki et al., 2007). However, cerebral vascular resistance in flight remains globally stable (Arbeille et al., 1995, 2001).

Spaceflight associated neuro-ocular syndrome represents an issue for future long-term manned missions. SANS manifestations include fundus anomalies like optic disk edema, globe flattening, choroidal and retinal folds, nerve fiber layer infarcts (cotton wool spots), and hyperopic refractive error shifts (Lee et al., 2018). SANS can’t be purely attributed to vascular problems. Current paradigm relates SANS rather to cephalad and orbital fluid transfer and chronic changes in intracranial pressure (Lee et al., 2018). The possible role of lymphatics and the venous system in SANS is under study. Indeed, microgravity-induced upward fluid redistribution induces jugular vein congestion (Herault et al., 2000; Arbeille et al., 2001).

##### Renal circulation

Similarly to brain, renal hemodynamics is characterized by high flow rate, is highly auto-regulated and not much affected by physical inactivity. Renal blood flow consists ∼20% of cardiac output at rest for kidneys mass of ∼0.4% body weight, to enable sufficient glomerular filtration rate of ∼180 L/day for precise regulation of fluid-volume homeostasis. In spaceflight renal plasma flow is stable, glomerular filtration rate is stable or increased, and filtration fraction tends to increase (Kramer et al., 2001). Thus we don’t expect substantial microgravity-induced renal vascular dysfunction.

#### Endothelium-Dependent Vasodilation

Many physical inactivity studies show impaired endothelium-dependent vasodilation of microvessels, contrary to that observed for conduit arteries. Reduced reactive post-occlusion hyperemic responses are observed at the leg level after 6-h sitting (Restaino et al., 2015), at the forearm level after 14-day HDBR (Shoemaker et al., 1998), and at both leg and forearm levels after 5-day horizontal bed rest (Hamburg et al., 2007). Fourteen-day HDBR diminishes forearm skin vasodilation in response to heating (Crandall et al., 2003). Attenuation of endothelium-dependent vasodilation, measured using laser Doppler coupled with acetylcholine (ACh) iontophoresis, was observed in the calf skin after 2-month HDBR in men (Yuan et al., 2015) and women (Demiot et al., 2007) and after 7-day dry immersion (Navasiolava et al., 2010), as well as in forearm skin after 6-month confinement (Yuan et al., 2019). Similarly, Hesse et al. (2005) found reduced dilation in response to ACh assessed by plethysmography at the forearm level after 2-week HDBR.

**FIGURE 1:**
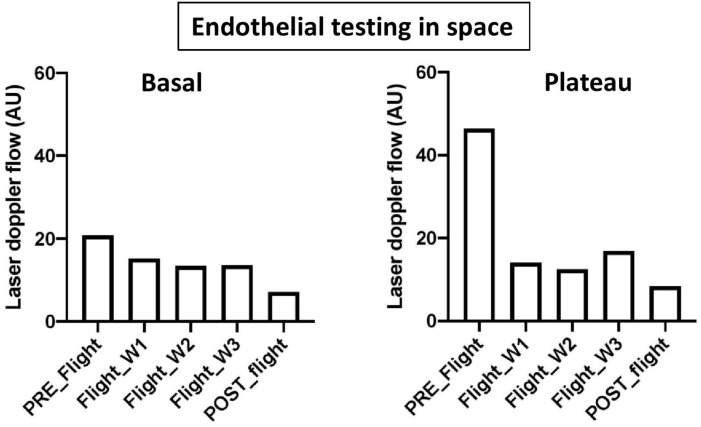
ACh testing with iontophoresis at the forearm skin level in two astronauts during the Tiangong -2 3-week space flight (W1, W2, W3). Basal, skin blood flow before ACh stimulation; Plateau, plateau phase of endothelium-dependent vasodilation after ACh stimulation. Data are mean values.

Regarding flight findings, during the Tiangong-2 Chinese 3-week space mission, we assessed basal skin blood flow and endothelium-dependent vasodilation at the forearm level using laser Doppler flowmetry coupled with ACh iontophoresis (integrated in the Cardiospace system, CNES/ACC; for technical details, see Li et al., 2019). During and after flight, we observed a slight decrease in basal skin blood flow and an almost complete absence of ACh-induced vasodilation at the plateau phase, indicating endothelial impairment (Figure 1).

#### Endothelium-Independent Vasodilation

Microcirculatory smooth muscle vasodilator function appears to be preserved after physical inactivity. Endothelium-independent vasodilation in response to the NO donor sodium nitroprusside (SNP) remains unchanged at the arm resistance vessel level after 13 days of HDBR (Hesse et al., 2005) and at the skin microcirculatory level after 2 months of HDBR (Demiot et al., 2007) or 7 days of dry immersion (Navasiolava et al., 2010). Dilation in response to incremental SNP infusion at the leg resistance vessel level is not altered by 4-week leg suspension (Bleeker et al., 2005c). Also, NO contribution to baseline vascular tone as estimated by NO synthase blockage is not altered by 6-week forearm cast (Green et al., 1997) or 4-week leg suspension (Bleeker et al., 2005c).

These studies suggest that the global increase in vascular resistance induced by physical inactivity is not necessarily explained by an impairment of NO dilator pathways. Unlike conduit arteries, for which endothelium-dependent vasodilation predominantly occurs via the NO pathway, three vasodilative pathways (i.e., NO, prostaglandin, and EDHF) are important for resistive vessels. Thus, at the microcirculatory level prostaglandin or EDHF pathways should be taken into account also, as well as neurovascular interactions.

#### Circulating Markers of Endothelial State

**FIGURE 2:**
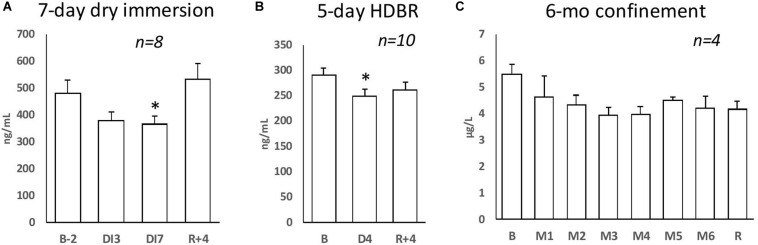
Plasma level of sCD146 during **(A)** 7-day dry immersion and **(B)** 5-day HDBR. **(C)** Serum level of sCD146 during 6-month confinement. Data are mean ± SEM; *p < 0.05 vs B.

Endothelial state markers are also impaired by physical inactivity. Data are summarized in Table 6. The number of circulating endothelial cells is increased after 2-month HDBR (Demiot et al., 2007). Circulating endothelial microparticles of an apoptotic phenotype increase after 5-day step limitation (Boyle et al., 2013), 7-day dry immersion (Navasiolava et al., 2010), and 60-day HDBR (Yuan et al., 2015). Some circulating angiogenic cell populations are reduced after 10-day limitation of high-intensity exercise in athletic individuals (Guhanarayan et al., 2014). Of note, endothelial glycocalyx, as assessed by blood levels of its components, is not altered after 5-day HDBR (Feuerecker et al., 2013). After 7-day dry immersion, the plasma level of VEGF decreases, whereas that of soluble E-selectin is unchanged, suggesting a decrease in antiapoptotic tone rather than inflammatory activation (Navasiolava et al., 2010). Soluble CD146, an endothelial molecule that appears to be involved in permeability and angiogenesis, is slightly decreased under 6-month confinement, 7-day dry immersion, and 4-day HDBR (Yuan et al., 2019) (Figure 2). These markers are likely mainly derived from the microvasculatory endothelium, considering that it is more extensive and more sensitive to physical inactivity than endothelium from large vessels.

**TABLE 6:** Circulating markers of endothelial state: effects of experimental models and countermeasures.

Variable	Inactivity model	Duration	Subjects Ctrl/CM	Estimated inactivity effect (without CM)	CM tested	Estimated CM effects	References
Circulating endothelial cells	HDBR	56 d	8F/8F	↑ (From 3.6/ml to 10.6/ml)	LBNP + aerobic + RE	Completely prevented	Demiot et al. (2007)
EMPs CD31+/CD42b-(“apoptotic”)	<5,000 steps/d	5 d	11M	↑ (From 18/μL to 104/μL)	_	_	Boyle et al. (2013)
EMPs CD31 + CD41-(“apoptotic”)	Dry immersion	7 d	8M	↑ (From 42/μL to 65/μL)	_	_	Navasiolava et al. (2010)
EMPs CD31+/CD42b-(“apoptotic”)	HDBR	60 d	7M/7M	↑ (From 46/μL to 97/μL)	CHM	Completely prevented	Yuan et al. (2015)
EMPs CD62E+(“activated”)	<5,000 steps/d	5 d	11M	No effect	_	_	Boyle et al. (2013)
Colony-forming unit-circulating angiogenic cells	Restraint from high-intensity exercise (athletes)	10 d	8M	↓36%	_	_	Guhanarayan et al. (2014)
CD34+ hemopoietic circulating angiogenic cells	Restraint from high-intensity exercise (athletes)	10 d	8M	No effect	_	_	Guhanarayan et al. (2014)
Components of endothelial glycocalyx	HDBR	5 d	12M crossover	No effect	Centrifugation	no effect	Feuerecker et al. (2013)
Soluble VEGF	Dry immersion	7 d	8M	↓27%	_	_	Navasiolava et al. (2010)
Soluble CD62E	Dry immersion	7 d	8M	No effect	_	_	Navasiolava et al. (2010)

*CHM, Chinese herbal medicine; CM, countermeasure; RE, resistive exercise; EMPs, endothelial microparticles; F, female; M, male*

### Venous Circulation

Venous compartment has a capacitance reservoir function contributing to cardiac output control. Venous functions characteristics are complex, and include filling/emptying properties, muscular pump efficiency, microvascular filtration. Microgravity-induced venous alterations represent an important issue worth an extensive review paper. Here we discuss venous changes only briefly.

#### Jugular Veins

In 2019 the first episode of blood clotting in space has been reported (Marshall-Goebel et al., 2019). Venous thromboembolism in space is life threatening and potentially a mission critical risk. In a cohort of 11 ISS astronauts, a half had stagnant or retrograde flow in the internal jugular vein at d50, and 1 crew member developed an occlusive internal jugular vein thrombus. Thus, weightlessness is associated with abnormal and stagnant cerebral venous outflow, which may lead to thrombosis in otherwise healthy astronauts (Marshall-Goebel et al., 2019).

#### Lower Limb Veins

Venous diseases of the lower extremities are very common, affecting about 25% of adults in westernized societies, with the spectrum ranging from simple telangiectasias to venous ulcerations. In microgravity context, leg venous function is important, as increased venous compliance and altered filling/emptying contribute to post-flight orthostatic intolerance. Venous occlusion air plethysmography is the main functional tool to assess limb venous functions. Studies with this method have shown that filling function of veins is altered during simulated weightlessness and spaceflight (Louisy et al., 1997; Fortrat et al., 2017). Several factors may be involved: muscular atrophy, changes in body fluids, decrease in venous tone, increase in venous compliance and changes at the microcirculatory level. Ultrasound measurement of venous cross-sectional area is a direct method to study main veins. Cross-sectional area increase in standing position after 90-day HDBR is greater in intolerant subjects (Belin de Chantemèle et al., 2004).

#### Splanchnic Level

Following 2-mo HDBR, baseline portal flow (proxy measured by portal vein cross-sectional area) diminishes by 19 ± 13%, related to decrease in blood volume. Splanchnic vasoconstrictive response to orthostatic stimulus (LBNP −45 mmHg), measured at portal vein level, is also decreased; moreover, insufficient flow reduction in splanchnic area is associated with orthostatic intolerance (Arbeille et al., 2008). These findings are in line with data of Behnke et al. (2013), showing impaired vasoconstriction of mesenteric veins in mice following 13–15-day flight.

## Mechanisms of Vascular and Microvascular Changes

### Mechanical Factors as Mechanisms of Vascular and Microvascular Changes (Figure 3)

#### Shear Stress

Physical inactivity decreases tissue demands and is associated with a general decrease in blood flow and, hence, shear stress. This is particularly the case for lower limbs, which are extremely unloaded in our models compared with normal daily activity. Langille and O’Donnell (1986) were the first to show, using a rabbit model of unilateral external carotid artery ligation, that a decrease in blood flow for 2 weeks causes inward remodeling. This response is abolished when the endothelium is removed, indicating that chronic changes in shear stress mediate endothelium-dependent vascular remodeling. Despite comparatively low magnitude of shear stress (0.5–5 Pa vs. more than 1,000 Pa for circumferential stretch of vessel wall during cardiac cycle), this force governs vascular remodeling. Sensitivity of vessel to shear stress seemingly depends on abundance of VEGFR3 (Baeyens and Schwartz, 2016).

**FIGURE 3:**
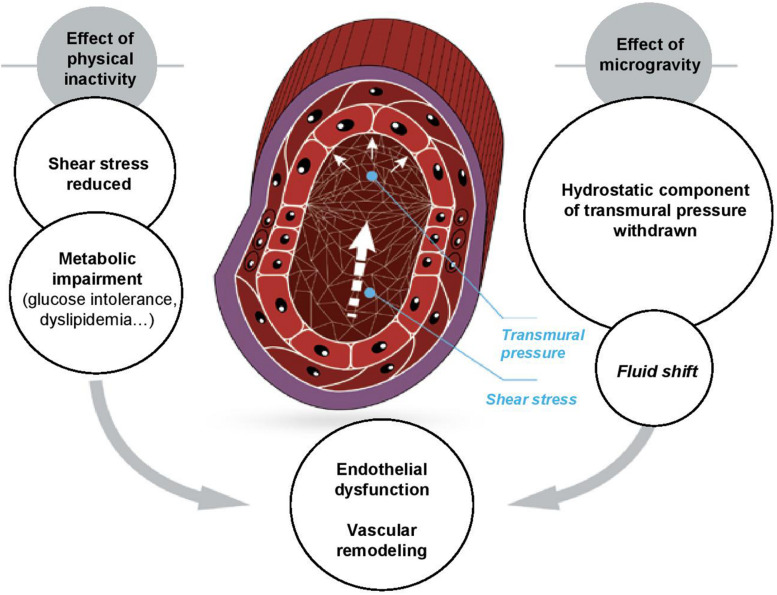
Vessels and mechanical forces in microgravity.

Inward remodeling of large vessels is believed to homeostatically regulate wall shear (Thijssen et al., 2010). A chronic shear stress diminution related to inactivity is particularly damaging to microcirculation (Boisseau, 2005). Differences in adaptation to an initial decrease in shear stress might underpin the differences in level of dysfunction for macro- and microcirculation. Whereas large vessel function is finally preserved, small vessels keep functional impairment.

#### Transmural Pressure

Vascular morphology and function, especially for smooth muscle cells, are sensitive to transmural pressure, which itself depends on blood and hydrostatic pressure. Systemic blood pressure is preserved or slightly decreased inflight (Norsk, 2019) and is not significantly modified by long-term bed rest (Fortney et al., 1996; van Duijnhoven et al., 2010a, b). However, hydrostatic pressure should be taken into account for vascular remodeling related to actual or simulated microgravity. Daily orthostatic stimulation induces large variations in hydrostatic pressure in the upper (up to −40 mmHg) and lower (up to +100 mmHg) parts of the body (Hargens and Watenpaugh, 1996). Hargens and Watenpaugh (1996) suggested that adaptation of vascular structure to microgravity is related to removal of the hydrostatic component (Zhang, 2001).

### Metabolic Factors as Mechanisms of Vascular and Microvascular Changes

Several studies demonstrate that even short periods of physical inactivity (e.g., bed rest for 3-7 days, dry immersion for 5-7 days) increase fasting blood insulin (Blanc et al., 2000; Hamburg et al., 2007; Navasiolava et al., 2011; Coupe et al., 2013; De Abreu et al., 2017), impair glucose tolerance (Blanc et al., 2000; Smorawinski et al., 2000; Hamburg et al., 2007; De Abreu et al., 2017), and alter lipid profile (Hamburg et al., 2007; Navasiolava et al., 2011; Coupe et al., 2013; De Abreu et al., 2017). Similarly, a 6-month flight increased insulin resistance index and glycated albumin level, did not alter significantly lipid profile, and inconsistently affected markers of inflammatory and oxidative stress (Hughson et al., 2016).

These metabolic abnormalities are associated with endothelial dysfunction at the microvascular level, as shown after 5-day bed rest (Hamburg et al., 2007), possibly by triggering several oxidative and pro-inflammatory pathways (e.g., increased reactive oxygen species production, activation of protein kinase C- and advanced glycation end product-induced pro-inflammatory signaling), leading to an unbalanced release of endothelial mediators. Of these mechanisms, increased oxidative stress seems to be the pivotal alteration (Potenza et al., 2009).

### Inflammatory Factors and Oxidative Stress as Mechanisms of Vascular and Microvascular Changes

The question of whether physical inactivity itself induces inflammation and increases oxidative state remains unanswered. Although acute exercise can induce oxidative stress, it also appears necessary for upregulating endogenous antioxidant defenses (Bloomer, 2008). Physical inactivity might promote an inflammatory state indirectly via metabolic changes. For example, lipid modifications are associated with altered levels of circulating cytokines and adipocytokines (Petersen and Pedersen, 2005). However, in the context of acute physical inactivity, there is generally no change in circulating inflammatory markers, arguing against the presence of systemic inflammation in these models. Hamburg et al. (2007) noticed that after 5-day non-strict bed rest, metabolic changes (i.e., insulin resistance and dyslipidemia) are not accompanied by changes in systemic inflammatory markers (i.e., C-reactive protein, interleukin (IL)-6, and tumor necrosis factor receptor-II). Similarly, 5-day strict HDBR does not alter inflammatory parameters (i.e., white blood cell number and counts, proinflammatory cytokine IL-6 and IL-8 levels, innate and adaptive immune responses) (Feuerecker et al., 2013). Dry immersion for 5–7 days does not modify C-reactive protein level (Coupe et al., 2013; De Abreu et al., 2017) or white blood cell number (Kozinets et al., 1983; Navasiolava et al., 2010). However, long-duration spaceflight (>140 days) triggers a hyper-inflammatory and aging immune phenotype (i.e., “inflammaging”) that appears to be related to inflight chronic stress, which may expose astronauts to risks for hypersensitivity diseases, such as allergies or autoimmune diseases (Buchheim et al., 2019). In 126–340-day spaceflight (*n* = 13) biomarkers of oxidative stress and inflammation increase inflight, but mostly restore within 1 week post-flight (Lee et al., 2020).

## Countermeasures and Their Effects on Macro- and Microcirculation

In this review we’ve chosen to discuss only countermeasures with potential cardiovascular effect, already tested using microgravity analogs (details on these countermeasures are summarized in Supplementary Data 1), or hypothetically applicable in microgravity context.

### Physical Countermeasures

#### Exercise

##### Aerobic exercise

Several studies show that daily physical activity and arterial stiffness are inversely correlated (O’Donovan et al., 2014) and that aerobic exercise decreases arterial stiffness (Tanaka et al., 1998; Hayashi et al., 2005). Regular aerobic exercise improves endothelial vasodilatory capacity, which is impaired by aging, metabolic problems, and hypertension. For example, DeSouza et al. (2000) demonstrated that endurance-trained men show no age-related decline in endothelium-dependent vasodilation. Moreover, in middle-aged individuals, aerobic exercise (i.e., walking) for 3 months restores the vasodilatory function loss observed in sedentary counterparts (DeSouza et al., 2000). Similarly, daily 40-min aerobic exercise for 3 months via a home-based aerobic exercise-training program appears to improve endothelium-dependent vasodilation in overweight adults independently of changes in body mass or composition (Mestek et al., 2010). A regular aerobic exercise program for 3 months (5–7 times a week) is also effective in improving endothelium-dependent vasodilation in both normotensive and hypertensive individuals (Higashi et al., 1999). Cutaneous vasodilation during exercise, as measured by laser Doppler, is impaired after 13-day HDBR (Lee et al., 2002) or 115-day flight (Fortney et al., 1998). Cutaneous vasodilation in response to forearm heating as estimated by plethysmography is also impaired after 13 days of HDBR (Crandall et al., 2003), whereas aerobic exercise (daily supine cycling for 90 min) prevents this skin microcirculatory impairment (Crandall et al., 2003; Shibasaki et al., 2003).

##### Resistive exercise

Resistive exercise is another type of physical exercise that may be beneficial for patients with CVD (Braith and Beck, 2008). Compared with aerobic exercise, however, the effects of resistive exercise on vascular function are more controversial. Resistive training was initially contraindicated for patients with coronary artery disease but now appears to be safe for clinically stable patients. Resistive training prevents age-associated declines in skeletal muscle mass and function (Hurley and Roth, 2000), but the effect of resistive training on exercise capacity is more disputed. Some studies show an increase in VO_2_ max after resistive training in patients with chronic heart failure, whereas others report no improvement. A study of patients with type 2 diabetes (Kadoglou et al., 2012) shows that resistive training does not affect VO_2_ max, lipid profile, or body fat but improves glycemic control and basal insulin level. A meta-analysis of randomized controlled trials between 1980 and 2011 determined that high-intensity resistance training is associated with increased arterial stiffness in young individuals with low baseline stiffness, whereas no such association is observed for moderate-intensity resistance training (Miyachi, 2013). In 60-day HDBR, resistive exercise completely prevents increases in carotid and femoral IMT and partially preserves femoral FMD but does not prevent a decrease in femoral diameter (van Duijnhoven et al., 2010a, b).

##### High-intensity interval training

High-intensity interval training (HIIT) comprises short bouts of maximal-intensity exercise alternated with less intense recovery intervals. HIIT is now considered a potential inflight countermeasure (Hurst et al., 2019). A 2014 meta-analysis of randomized trials determined that HIIT is more effective in improving brachial artery vascular function than moderate-intensity continuous training, perhaps due to its tendency to positively influence cardiorespiratory fitness, traditional CVD risk factors, oxidative stress, inflammation, and insulin sensitivity (Ramos et al., 2015). Similarly, a recent study of healthy inactive adults shows that 12-week HIIT is more efficient in improving FMD and decreasing arterial stiffness than 12-week moderate continuous training (Ramírez-Vélez et al., 2019). In 60-day HDBR, high-intensity resistance training involving reactive jumps mitigates cardiovascular deconditioning (although arterial and microcirculatory state were not specifically assessed) (Maggioni et al., 2018). Resistive training impairs endothelial function as evidenced by decreased FMD, presumably due to a sustained elevation in blood pressure; however, high-intensity resistance exercise with low repetitions, which minimizes barostress on vasculature, maintains endothelial function (Morishima et al., 2018).

##### Combined resistive and aerobic exercise and endothelium

Combined resistive and aerobic exercise improves endothelium-dependent vasodilation (Maiorana et al., 2000). Demiot et al. (2007) showed that during 60-day HDBR, endothelium-dependent vasodilation and the number of circulating endothelial cells are preserved in individuals who engage in resistive exercise (i.e., flywheel) and aerobic exercise (i.e., treadmill) coupled with LBNP, indicating the protection of endothelial function.

#### Whole Body Vibration

Whole body vibration has been proposed as a therapeutic tool for many years. Numerous studies show WBV beneficial effects for bone mass (Gomez-Cabello et al., 2012), neuromuscular function (Bosco et al., 1999; Torvinen et al., 2002; Fontana et al., 2005), and the endocrine system (Di Loreto et al., 2004). Some studies suggest that WBV acutely decreases arterial stiffness. Specifically, Otsuki et al. (2008) showed that 10 sets of vibration (26 Hz) for 60 s in a static squat position decreases brachial-ankle pulse wave velocity, an index of arterial stiffness, immediately after the WBV trials, with a return to baseline within 60 min. In inactivity models, a combination of vibration and exercise has a beneficial vascular effect. In an experiment with 60-day HDBR (i.e., the second Berlin Bed Rest study), van Duijnhoven et al.(2010a,b) compared resistive exercise alone and resistive exercise combined with WBV and found that combined countermeasures completely preserve superficial femoral artery FMD and partially preserve its diameter, whereas resistive exercise alone is not sufficient to counteract vascular changes. These results are in accordance with those of the first Berlin Bed Rest study, in which combined resistive exercise and WBV attenuated the decrease in femoral diameter induced by 52 days of horizontal bed rest and preserved femoral FMD after 24 days but not 52 days of bed rest (Bleeker et al., 2005b). The mechanisms involved in vascular protection by WBV remain unknown but could include immediate increases in femoral and popliteal artery blood flow and shear rate (Kerschan-Schindl et al., 2001).

#### LBNP

In HDBR studies, the effects of LBNP as a countermeasure were tested alone (Güell et al., 1991) and in association with exercise (Arbeille et al., 1995, 2008). LBNP countermeasure alone, with several sessions per day, mitigated orthostatic hypotension in response to tilt-test at the end of 30-day HDBR (Güell et al., 1991). A combination of LBNP with exercise preserved leg vasoconstrictive response to orthostatic stimulus following 1-month HDBR in men and 2-month HDBR in women, as measured by Doppler ultrasound, while in control subjects this vasoconstrictive response was decreased (Arbeille et al., 1995, 2008). Besides, this combined LBNP + exercise countermeasure prevented endothelial impairment induced by 2-month HDBR in women (Demiot et al., 2007). Today, LBNP can be used in combination with many countermeasures such as fluid loading (i.e., salt and water), nutrition supplementation, and exercise. With recent occurrence of thrombotic episode in flight, utility of LBNP countermeasure to counteract headward fluid shift and improve jugular blood flow patterns is discussed (Marshall-Goebel et al., 2019).

#### Artificial Gravity

Artificial gravity is a promising countermeasure that reproduces terrestrial conditions (Evans et al., 2018) and could be combined with exercise or vibration (Clement and Pavy-Le Traon, 2004). Even short intermittent 1-G exposure may suffice to prevent adverse effects of microgravity (Zhang, 2001). The effect of centrifugation on vascular function would also be interesting to study. We speculate that gravity reproduction could be beneficial for the cardiovascular system. However, gravity at the lower limb level is much greater in a short arm centrifuge, with potential negative effects to microcirculation. Studies specifically examining the vascular and microcirculatory consequences of artificial gravity are still lacking. However in a murine model, apoptosis of retinal vascular endothelial cells induced by 35-day spaceflight was mitigated by continuous 1-g artificial gravity (Mao et al., 2018).

### Nutritional and Pharmacological Countermeasures

#### Caloric Restriction

Caloric restriction is a dietary intervention that maintains proper nutrition but reduces caloric intake. Caloric restriction might be beneficial for the cardiovascular system, even in healthy non-obese individuals. Lefevre et al. (2009) showed that 25% caloric restriction for 6 months (i.e., the CALERIE trial) decreased estimated 10-year CVD risk by 29%, although there was no effect on endothelial function as assessed by FMD at the brachial artery level. However, Hesse et al. (2005) demonstrated that 25% caloric restriction for 13 days, mainly achieved by reducing fat intake to a minimum recommended level of 60 g/day, improves the response of forearm resistance vessels to ACh.

#### Polyphenols and Other Natural Extracts

Polyphenols are organic, mainly natural substances characterized by the presence of several phenol structural units. They include simple phenols, flavonoids, and non-flavonoids such as stilbenes (e.g., resveratrol), saponin, curcumin, and tannins. Potential use of different polyphenols in prevention and treatment of CVD is reviewed in a recent paper of Giglio et al. (2018). Epidemiological studies show an inverse relationship between dietary polyphenol consumption and mortality from CVD (Middleton et al., 2000). Polyphenols exert numerous biological effects that might protect the cardiovascular system, including vasodilatory, antioxidant (Andriantsitohaina et al., 2012), anti-aggregatory, and cholesterol-lowering (Ngamukote et al., 2011) effects. Polyphenols may improve endothelial function. Polyphenol-rich products at relatively low doses, corresponding to two glasses of red wine or daily consumption of 46 g dark chocolate for 2 weeks, increase FMD in healthy individuals. Similarly, polyphenol-rich products as black tea, green tea, and red grape extracts improve FMD in individuals with coronary artery disease (Andriantsitohaina et al., 2012). Nutritional supplements rich in polyphenols such as chocolate (Garcia et al., 2018) and walnut extract (Papoutsi et al., 2008) could also have potential effects on blood vessels.

Resveratrol is a natural polyphenol synthesized by some plants. In particular it is contained in red wine. In human studies, acute resveratrol prescription improves FMD in overweight and mildly hypertensive individuals as soon as 1 h after consumption (Wong et al., 2011). Prescription of modified resveratrol for 3 months improves endothelial function in adults with metabolic syndrome (Fujitaka et al., 2011). In 60-day HDBR antioxidant/anti-inflammatory cocktail containing resveratrol and other polyphenols, vitamin E, selenium, and omega-3, was not efficient to prevent muscle mass and strength loss (Arc-Chagnaud et al., 2020). However, due to its pleiotropic effects, resveratrol may be a good candidate for correcting cardiovascular alterations.

Chinese herbal medicine is one of the most important modalities of traditional Chinese medical care. Chinese herbal medicine countermeasures against vascular deconditioning are attractive due to their pleiotropic effects that are not limited to a single mechanism or a single application point (Lu et al., 2004). In 60-day HDBR, TaikongYangxin (“outer space heart-nourishing”), a herbal formula created by the Chinese space agency to boost the physical conditions of astronauts and improve their adaptability in an extreme environment, contributes to the prevention of post-bedrest loss of vasoconstriction in leg and splanchnic areas (Yuan et al., 2012), improves microvascular endothelial function (decrease in plateau vasodilative response to ACh from 46 to 31% of maximal vasodilation to heating, induced by 60-day HDBR, was completely prevented) and preserves endothelial integrity (increase in “apoptotic” EMPs induced by 60-day HDBR was prevented) (Yuan et al., 2015). This herbal extract is composed of over 10 ingredients including Panax ginseng, Astragalus membranaceus, Ligusticum wallichii, Schisandra chinensis, Ophiopogon japonicas, Rehmania glutinosa, Drynaria fortunei, and Poria cocos. Several active components of this formula may be capable of ameliorating endothelium-dependent vasodilation with potential synergic interactions.

#### Medications

Unexpectedly, medications are almost not applied to protect vascular functions. Although midodrine (Platts et al., 2004) has been proposed to promote vasoconstriction after spaceflight and avoid orthostatic hypotension, its interactions with anti-emetics used in this context have interrupted the studies with this compound.

## Conclusion and Perspectives

Actual and simulated weightlessness causes both structural and functional vascular changes. Although a chronic decrease in shear stress due to physical inactivity appears to be the main contributing factor, metabolic and circulating factors should also be taken in account. Fluid shift-related changes in hydrostatic pressure seem to be less important on the arterial side.

Studies of vascular properties, although well developed (diameter, IMT, compliance and flow rate measurements for large vessels; vasodilation capacity assessment using ischemic stimulus, heating, ACh and NO-donors; some biological assays), explore the vessels only partially. There are many unresolved questions about vascular changes induced by physical inactivity. Vasodilation by prostaglandin pathways, microcirculatory neurovascular interactions, and endothelial changes at the organ level (e.g., muscles, bones, and brain) and their potential links to local oxidative stress are some areas that should be explored.

To date, countermeasures based on physical exercise remain most effective against vascular dysfunction induced by physical inactivity and space environment. Exercise modalities were recently extensively discussed in the *Frontiers* research topic “Optimization of exercise countermeasures for human space flight – lessons from terrestrial physiology and operational considerations” (Scott et al., 2020). Resistive exercise and vibration could provide additional benefits. Specialized diets and nutritional supplements are also very promising, particularly plant extracts and hypocaloric or lipid-depleted diets that could preserve endothelial function.

Furthermore, with deep space missions beyond Earth’s protective magnetosphere, irradiation factors become other major contributors to cardiovascular (i.e., endothelial) impairment (Delp et al., 2016; Hughson et al., 2018), which implies the importance of antioxidants, nutraceuticals, and radiation shielding in a countermeasure program in addition to physical fitness.

## Author Contributions

All authors contributed in drafting and revising of this review manuscript.

## Conflict of Interest

The authors declare that the research was conducted in the absence of any commercial or financial relationships that could be construed as a potential conflict of interest.
