# Supplementary material for: Vascular and Microvascular Dysfunction Induced by Microgravity and Its Analogs in Humans: Mechanisms and Countermeasures
Source: Front Physiol. 2020 Aug 20;11:952. doi: 10.3389/fphys.2020.00952 (PMC7468431; doi:10.3389/fphys.2020.00952)
Supplement: Supplementary file 1 [file Table_1.pdf]

Supplementary Data 1. Countermeasures tested in our experimental models

Countermeasure	Regime	Inactivity model	Reference
Contralateral leg	Non-casted leg	7-d Unilateral leg cast	Sugavara et al., 2004
Contralateral leg	Non-suspended leg	28-d Unilateral leg suspension	Bleeker et al., 2005a
Non-casted forearm controls	Subjects matched in age and activity level	42-d Forearm cast	Green et al., 1997
Usual daily activity	Usual level of physical activity	5-d Horizontal BR	Hamburg et al., 2007
Usual daily activity	Sex and age matched controls, usual activity	7-d HDBR	Bonnin et al., 2001
3 bouts of 5-min walking	Walking on treadmill for 5 min at 2 mph at min 30, min 90 and min 150	3-h Sitting	Thosar et al., 2015
1 bout of 10-min walking	10-min walk at self-selected pace (~1000 steps) following 6h of uninterrupted sitting	6-h Sitting	Restaino et al., 2015
Unilateral fidgeting bouts	One leg was subjected to intermittent fidgeting (1 min on/4 min off, ~250 taps/min); contralateral leg remained still	3-h Sitting	Morishima et al., 2016
Cycle ergometry	Supine cycling at 75% of max HR pre-HDBR, 90 min daily (2 bouts of 45min or 3 bouts of 30 min)	14-d HDBR	Crandall et al., 2003
LBNP+aerobic +resistive exercise	Once per 3 days flywheel ~30min; 3-4 times/wk treadmill at 40-80% VO2peak combined with 50-60mmhg LBNP for 35 min, then LBNP alone for another 10 min	56-d HDBR	Demiot et al., 2007
Resistive exercise	3 times/wk for 5–7 min/session, Galileo Space device specifically developed for application under microgravity and bedrest	60-d HDBR	Van Duijnhoven et al., 2010a,b
Resistive vibration exercise	Twice daily for 30 min (8 min pure exercise time), except for Sundays and Wednesday afternoons, Galileo Space device. Initial vibration frequency: 19 Hz. If subjects managed to increase exercise time, vibration frequency was increased.	52-d Horizontal BR	Bleeker et al., 2005b
Resistive vibration exercise	3 times/wk for 5–7 min/session, Galileo Space device; vibration at 16-26Hz	60-d HDBR	Van Duijnhoven et al., 2010a,b
Centrifugation	30 min daily or 6x5min daily at 1g at the center of mass	5-d HDBR	Feuerecker et al., 2013
Thigh cuffs	10h/day, counter pressure of 30mmHg	7-d HDBR	Arbeille et al., 1999
Unilateral foot heating bouts	One foot was submerged in 42°C water 3 times a day x 30 min; contralateral leg remained dry and non-heated	5-d less 5000 steps/d	Texeira et al., 2017
Low fat hypoenergetic diet	25% restriction in calories mainly by fat reducing to a minimum recommended level of 60 g/day	13-d HDBR	Hesse et al., 2005
Chinese medicine	6-g oral honeyed pill 3 times a day	60-d HDBR	Yuan et al., 2015
